# A successfully treated gastric mucormycosis in an immunocompetent patient: Case report and literature review

**DOI:** 10.1002/ccr3.7540

**Published:** 2023-06-17

**Authors:** Asma Salameh Albtoosh, Moayad Shaf'ei, Sa'ed Al Hayek, Lara Ibrahim Ramdan, Nisreen Abu Shahin, Mohammad Alzyoud, Randa Farah

**Affiliations:** ^1^ Department of Respiratory and Internal Medicine, School of Medicine The University of Jordan Amman Jordan; ^2^ Jordan University Hospital Amman Jordan; ^3^ Ministry of Health Amman Jordan; ^4^ Department of Internal Medicine, School of Medicine The University of Jordan Amman Jordan; ^5^ Department Pathology, School of Medicine The University of Jordan Amman Jordan; ^6^ Department of Nephrology and Internal Medicine School of Medicine, The University of Jordan Amman Jordan

**Keywords:** fungal infection, gastric biopsy, gastric mucormycosis, gastrointestinal bleeding, immunocompetent patient

## Abstract

Mucormycosis is an opportunistic fungal infection that usually affects patients with diabetes mellitus or immunosuppression. The fungus invades the nearby blood vessels leading to thrombosis and necrosis of the organs involved. Although Mucorales can invade any organ in the body, the gastrointestinal system is an uncommon site for infection. Mucormycosis is a fatal infection, and prompt intervention is required to ensure survival. In this report, we present a case of a 46‐year‐old man with history of valve replacement surgery on warfarin, who admitted with abdominal pain and life‐threatening gastrointestinal bleeding. Esophagogastroduodenoscopy revealed an active gastric ulcer bleeding, and the diagnosis of mucormycosis infection was confirmed with direct microscopy and histopathological evaluation from a tissue *biopsy*. Typically, antifungal therapy alone is inadequate to control mucormycosis infection and surgical intervention is often required. Our patient was successfully treated using antifungal therapy alone. This report presents a rare case of gastrointestinal mucormycosis in setting of valve replacement and was successfully treated with antifungal therapy.

## Graphical abstract

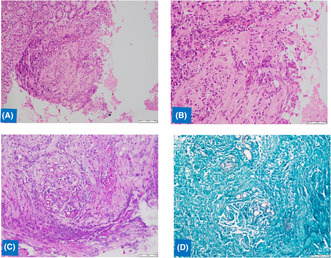

## 1 INTRODUCTION

Mucormycosis, an uncommon and possibly fatal fungal infection, is caused by fungus from the class *Mucorales*. Mucormycosis opportunistically affects patients with seriously weakened immune systems and diabetic ketoacidosis.^1^ Although this organism can affect any organ, it is most frequently isolated from the nasal sinuses, lungs, skin, and brain. Nevertheless, gastrointestinal mucormycosis involvement is very uncommon.^2^ The diagnosis of mucormycosis is usually made by histological testing and biopsy.^3^ Antifungal medications in combination with surgical intervention are needed to treat most mucormycosis patients.^4^ In this case report, we are presenting a rare case of gastric mucormycosis causes upper gastrointestinal bleeding.

## 2 CASE REPORT

A 46‐year‐old man with a significant medical history of mechanical aortic valve replacement in 2020 due to bicuspid valve, He is on on oral anticoagulant (warfarin) and the dose is alternating between 3 and 5 mg. He is actively smoking with a 40 pack‐year smoking history.

The patient was transferred to Jordan University Hospital (JUH), a tertiary care center in Jordan for evaluation of upper Gastrointestinal (GI) bleeding. Our patient initially presented to the primary hospital with severe abdominal pain, hematemesis, and melena. He was found to have low blood pressure and decreased level of consciousness. At the time, he was intubated and stabilized with intravenous and blood transfusions. Initial laboratory data revealed a hemoglobin (Hgb) level of 2 g/dL, an international normalized ratio (INR) of 10, and serum creatinine (SrCr) level of 11 mg/dL (his baseline is 1 mg/dL). Subsequently, he received a massive blood transfusion of 8 units of packed red blood cells, fresh frozen plasma and vitamin K. Our patient was stabilized with Hgb reaching 9 g/dL and INR at 3.5. Consequently, he was extubated and referred to JUH for further investigation of his underlying disease.

Upon arrival at the JUH emergency room, he was still complaining of epigastric pain and black tarry stool. On examination: his vital signs revealed a heart rate of 105 beats per minute, blood pressure of 106/70 mmHg, respiratory rate of 26, and oxygen saturation of 89% on room air. He looked very ill, pale but conscious and oriented to time, place, and person. He had elevated jugular venous pressure and bilateral lower limb edema. Moreover, upon auscultating the chest, he had a decreased air entry with prominent crepitation at the bases of the lungs. His abdominal examination showed mild epigastric tenderness without guarding or rigidity, and no evidence of organomegaly. Rectal examination (PR) revealed a black tarry stool with a small amount of fresh blood. His laboratory results upon admission to JUH showed serum Hb was 8.5 g/dL, SrCr was 11.9 mg/dL, and potassium was 6.08 mg/dL. Other laboratory results are shown in Table 1. His arterial blood gases showed a pH of 7.22, PCO_2_ of 26 mmHg, HCO3 of 10.6 mEq/L, and oxygen saturation of 97% on 3 L of oxygen. As a result, hemodialysis was initiated to stabilize the patient and correct his fluid overload and electrolytes disturbances. In addition, an upper GI endoscopy was performed, which revealed a 1.5 cm large, cratered ulcer with a slightly depressed center just below the gastroesophageal junction in the proximal gastric body along the greater curvature. The ulcer has a clean base with a visible vessel in the proximal margin with no active bleeding. The rest of the gastric mucosa is slightly nodular and hyperemic, a biopsy was taken from the ulcer and submucosal adrenaline injection, and a heating probe was used to the visible vessel. Subsequently, the patient underwent a lower endoscopy and a whole‐body CT scan, both of which were negative for other lesions.

**TABLE 1:** Laboratory tests upon Admission to Jordan university hospital.

Test	Value	Reference range
Hematocrit	25.6%	41%–50%
Hemoglobin (Hb)	8.4 g/dL	13.5–17 g/dL
Mean corpuscular volume (MCV)	82.9 fL	80–100 fL
Red blood cell distribution width (RDW)	19.8%	12%–15%
White blood cells (WBCs)	14,950 cell/μL	4500–11,000 cell/μL
Neutrophils	93.3%	55%–70%
Lymphocytes	2.2%	20%–40%
Monocytes	2.9%	2%–8%
Eosinophils	0.7%	1%–4%
Basophils	0.1%	0.5%–1%
Platelets count	165,000 per μL	150,000–450,000 per μL
Prothrombin time (PT)	39.3 s	11–13.5 s
INR	3.79	0.8–1.1
Partial thromboplastin time (PTT)	39.3 s	25–35 s
Random blood sugar (RBS)	90 mg/dL	<140 mg/dL
HbA1C	5.2%	<5.7%
Urine ketones	Negative	Negative
Chloride (Cl)	114 mg/dL	96–106 mg/dL
Potassium (K)	6.08 mg/dL	3.7–5.2 mg/dL
Calcium (Ca)	7 mg/dL	8.6–10.3 mg/dL
Phosphorous	9.3 mg/dL	2.8–4.5 mg/dL
Creatinine (Cr)	11.9 mg/dL	0.7–1.3 mg/dL
Urea	250 mg/dL	6–24 mg/dL
Albumin	2.85 g/dL	3.4–5.4 g/dL
C‐reactive protein (CRP)	79.9 mg/dL	0.3–1.0 mg/dL
Ferritin^a^	1189 ng/mL	12–300 ng/mL

^a^ It provide a more complete picture to the patient at the time of admission.

**FIGURE 1:**
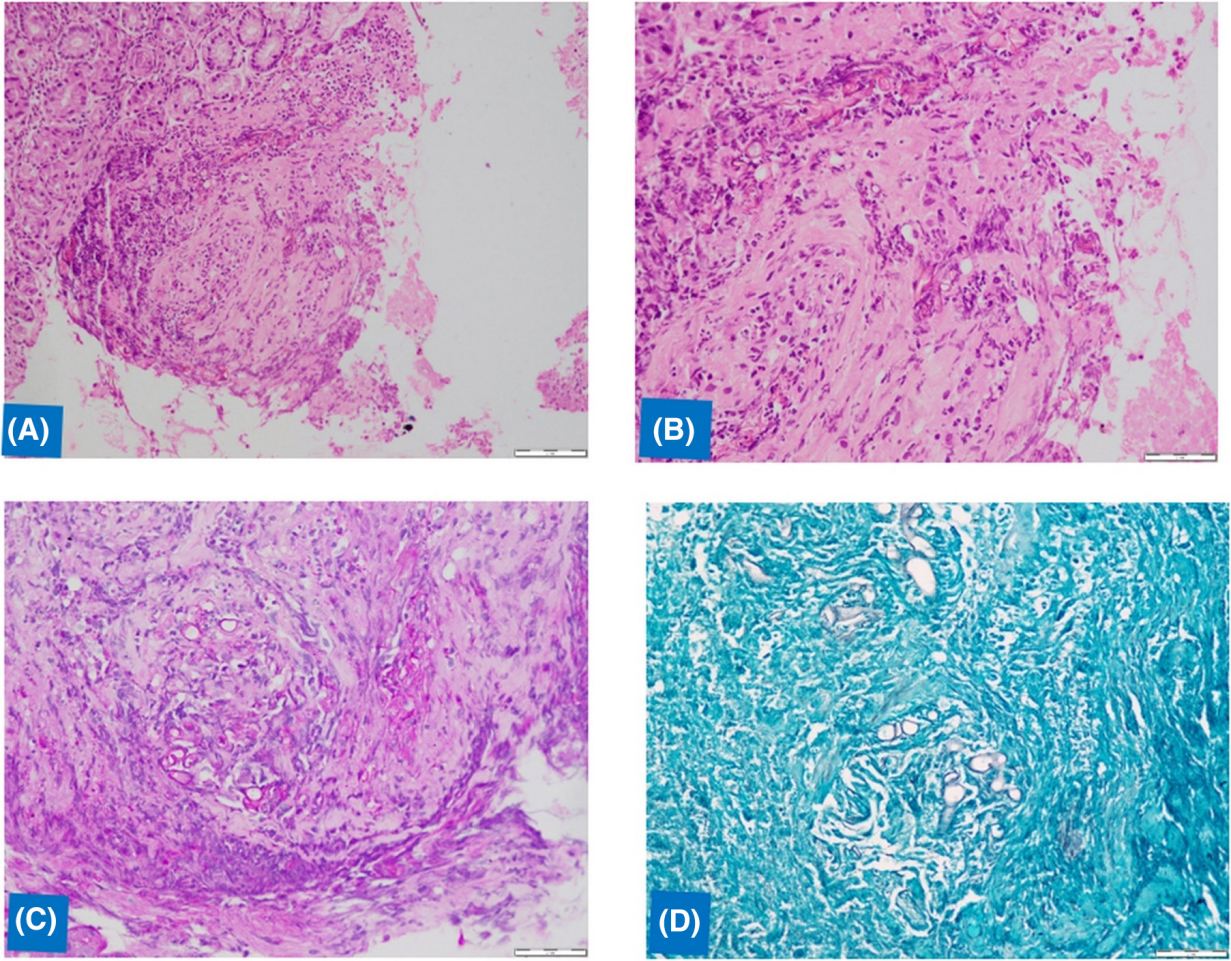
Histopathologic sections showing aseptate fungal hyphae consistent with mucormycoses invading the gastric mucosa (A and B: Hematoxylin and eosin stain, 20× and 40×, C: PAS stain, 40×, D: Gomori's methenamine–silver stain, 40×).

The histopathologic sections demonstrated a gastric ulcer infiltrated with wide and branching aseptate hyphae consistent with Mucormycosis and seen involving the vessels. Periodic Acid‐Schiff (PAS) and Gomori's methenamine–silver (GMS) special stains confirmed the fungal morphology (Figure 1A–D). Subsequently, the patient was started on antifungal therapy with liposomal amphotericin B (5 mg/kg/day) to complete a total of 4 weeks.

Over the following weeks, the patient's clinical condition significantly improved, and his upper GI bleeding was controlled with a stable Hb. Furthermore, the patient's renal function recovered, and we stopped dialysis after 3 weeks of admission. A follow‐up upper endoscopy, after 4 weeks of treatment with antifungal, was done and showed that the previous gastroesophageal junction ulcer had completely resolved, which indicates a successfully treated gastric mucormycosis.

Eventually, the patient was discharged with significant improvements in his clinical and functional status.

## 3 DISCUSSION

Mucormycosis is an acute opportunistic fungal infection that is caused by fungus from the class Mucorales. The most commonly isolated pathological species of *Mucorales* are *Rhizopus* followed by *Mucor*.^5^ In a systematic review and meta‐analysis of 851 cases of mucormycosis, rhino‐orbital‐cerebral mucormycosis (ROCM) involvement was most commonly observed among the reviewed cases (34%), followed by skin (22%) and lung involvement (20%). Gastrointestinal mucormycosis was reported only in 8% of the cases.^2^

Gastrointestinal mucormycosis is an unusual manifestation of mucormycosis infection which commonly affects the stomach, small intestines, and/or colon.^2^ Mucorales reach the gastrointestinal tract via ingestion of contaminated moldy food. Contaminated medical devices comprise another route of entry for the filamentous fungus. Subsequently, Mucorales invade the nearby blood vessels resulting in vessel thrombosis and tissue necrosis.^6^ This makes the presentation of gastric mucormycosis diverse. A literature review of 31 cases showed that gastric mucormycosis most commonly presented in middle‐aged male patients, with abdominal pain and GI bleeding being the most frequently encountered symptoms.^7^ This is consistent with our case, as the patient was a 45‐year‐old male patient who presented with abdominal pain and a life‐threatening GI bleed. The severity of the bleeding, significant Hgb drop, and concomitant acute kidney injury can be explained by the warfarin toxicity and pathogenic invasion of Mucorales into the blood vessels.

The vast majority of patients with mucormycosis have an underlying risk factor; these include diabetes mellitus, neutropenia, steroid use, trauma, and a history of malignancies.^2^ Despite some studies finding a relationship between hyperferritinemia and mucormycosis,^8^ it is not yet explained whether hyperferritinemia is a risk factor for mucormycosis^9^ or it is just an inflammatory marker. Most reported cases had received deferoxamine‐iron chelator, which has an essential role in the pathogenicity of Mucorales, after multiple chronic blood transfusions.^10^, ^11^ In the case described here, our patient was found to have hyperferritinemia a week after mucormycosis diagnosis, but he did not receive an iron chelator which adds to the controversy about the role of ferritin in the pathogenicity of the disease.^10^ It is unclear why an immunocompetent patient developed this fungal infection, but several cases in the literature described similar circumstances.^12^, ^13^, ^14^

Given the rarity of the disease and the variety of presenting symptoms, a high index of suspension is needed to early recognize and diagnose mucormycosis.^15^ Mucormycosis should be suspected early based on the risk factors, clinical manifestations, and imaging findings. Then, diagnosis is usually confirmed by microbiological, histopathological, and molecular techniques.^4^ In spite of the fact that imaging findings are nonspecific, they may aid in excluding disseminated disease and some complications.^16^ The visual appearance of the infection focuses during esophagogastroduodenoscopy (EGD) in previously reported cases showed patchy ulcers with varying degrees of exudates. This usually warrants a biopsy followed by histopathological examination and culture to exclude malignancies in the first place.^7^, ^17^, ^18^ Potassium hydroxide mount and hematoxylin and eosin stains may provide a rapid visualization of the fungal hyphae and give a presumptive diagnosis intraoperatively to define clear surgical margins. An organism that is thin‐walled, with ribbon‐like hyphae, with no or little septations, GMS, and PAS‐positive is consistent with *Mucorales*.^19^ There is emerging evidence that molecular tests and nucleic acid sequencing can help to individualize treatment based on the isolated species of Mucorales and their drug resistance profile.^20^ Early diagnosis of the disease is of utmost importance. Studies have shown that it decreases mortality, extensive surgical resection, and suffering.^21^

Gastric mucormycosis is a rapidly fatal condition, with a mortality rate reaching up to 54%.^2^ The paucity of cases in the literature makes the treatment of gastric mucormycosis challenging, as no specific guidelines were released. The treatment approach starts by reversing the risk factors and managing the underlying comorbidities as much as possible. The antifungal medication, namely liposomal amphotericin b (LAB), in combination with surgical intervention, is considered the first‐line treatment of mucormycosis.^4^ The liposomal formulation of amphotericin B is preferred by many experts due to its lower renal toxicities at higher doses. There are no clear data about the optimal dose and duration of the LAB treatment in mucormycosis, and treatment should be continued until the complete resolution of the infection. Despite animal experiments showing that the combination therapy of amphotericin B and echinocandins may improve the outcomes, additional research is yet needed to prove this hypothesis.^4^ Surgical debridement of the fungal focus is warranted, as the ulcerative, necrotic, and thrombotic infection focus of mucormycosis is poorly penetrated by LAB. However, a recently published review of 20 cases of gastric mucormycosis reported that the use of antifungal medication alone has resulted in the resolution of the disease in four cases out of six, while only 5 cases out of 10 were successfully treated using a combination of antifungal and surgery.^17^ This is in accordance with our case, as the patient was successfully treated using liposomal amphotericin b alone for 4 weeks until his EGD returned to normal. In conclusion, we presented a case of gastric mucormycosis in an immunocompetent patient who presented with massive GI bleeding. Biopsy with histological examination is essential for diagnosis. Mucormycosis is a catastrophic infection, and rapid intervention with the appropriate treatment is necessary for survival.

## AUTHOR CONTRIBUTIONS

**Asma Salameh Albtoosh:** Supervision; writing – review and editing. **Moayad Shaf'ei:** Data curation; investigation; writing – original draft. **Sa'ed Al Hayek:** Data curation; investigation; writing – original draft. **Lara Ibrahim Ramdan:** Data curation; investigation; writing – original draft. **Nisreen Abu Shahin:** Data curation; investigation; writing – original draft. **Mohammad Alzyoud:** Data curation; investigation; writing – original draft. **Randa Farah:** Supervision; writing – review and editing.

## FUNDING INFORMATION

The study was funded by the authors themselves.

## CONFLICT OF INTEREST STATEMENT

All authors declare that they have no conflict of interest.

## ETHICS STATEMENT

Verbal and written consents were obtained from the patient before writing the case or using investigations to participate.

## CONSENT

Written informed consent was obtained from the patient to publish this report in accordance with the journal's patient consent policy.

## Data Availability

The authors confirm that all relevant data to this case were included in the article. Further data will be available directly from the author upon request.
